# Supplementary material for: Atomic-level polarization in electric fields of defects for electrocatalysis
Source: Nat Commun. 2023 Nov 29;14:7849. doi: 10.1038/s41467-023-43689-y (PMC10686988; doi:10.1038/s41467-023-43689-y)
Supplement: Supplementary file 1 — Supplementary Information File [file 41467_2023_43689_MOESM1_ESM.pdf]

Supplementary Information for

Atomic-level polarization in electric fields of defects for electrocatalysis

Jie Xu[1,7], Xiongxiong Xue[2,7], Gonglei Shao[3]*, Changfei Jing[4], Sheng Dai[4], Kun He[1], Peipei Jia[5], Shun Wang[1], Yifei Yuan[1]*, Jun Luo[5]*, Jun Lu[6]*

[1] College of Chemistry and Materials Engineering, Wenzhou University, Wenzhou, Zhejiang 325035, China.

[2] School of Physics and Optoelectronics, Xiangtan University, Xiangtan 411105, China.

[3] Engineering Research Center of Advanced Functional Material Manufacturing of Ministry of Education, School of Chemical Engineering, Zhengzhou University, Zhengzhou 450001, China.

[4] Feringa Nobel Prize Scientist Joint Research Centre, School of Chemistry and Molecular Engineering, East China University of Science & Technology, Shanghai 200237, China.

[5] ShenSi Lab, Shenzhen Institute for Advanced Study, University of Electronic Science and Technology of China, Longhua District, Shenzhen 518110, China.

[6] College of Chemical and Biological Engineering, Zhejiang University, Hangzhou 310027, China.

[7] These authors contributed equally: Jie Xu, Xiongxiong Xue

*Correspondence to: shaogonglei@zzu.edu.cn; yifeiyuan@wzu.edu.cn; jluo@uestc.edu.cn; junzoelu@zju.edu.cn

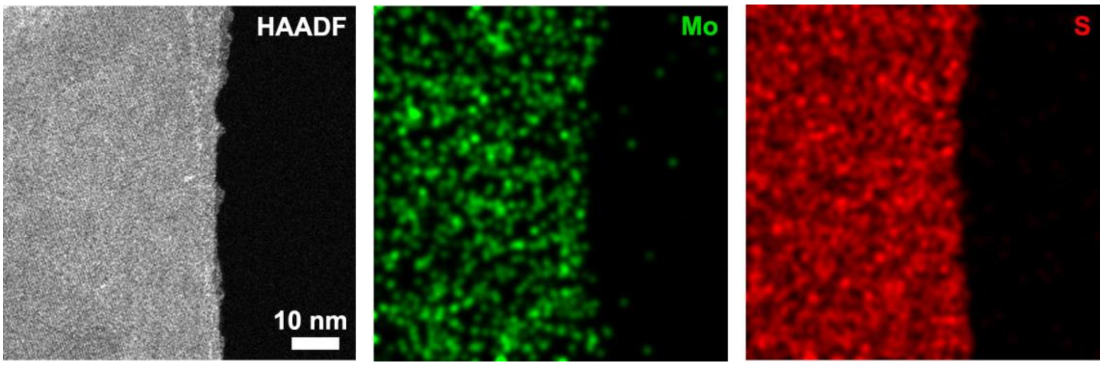

Supplementary Fig. 1 EDS mapping results of the pristine MoS_2, including the EDS mapping of Mo, S, and the corresponding HAADF image.

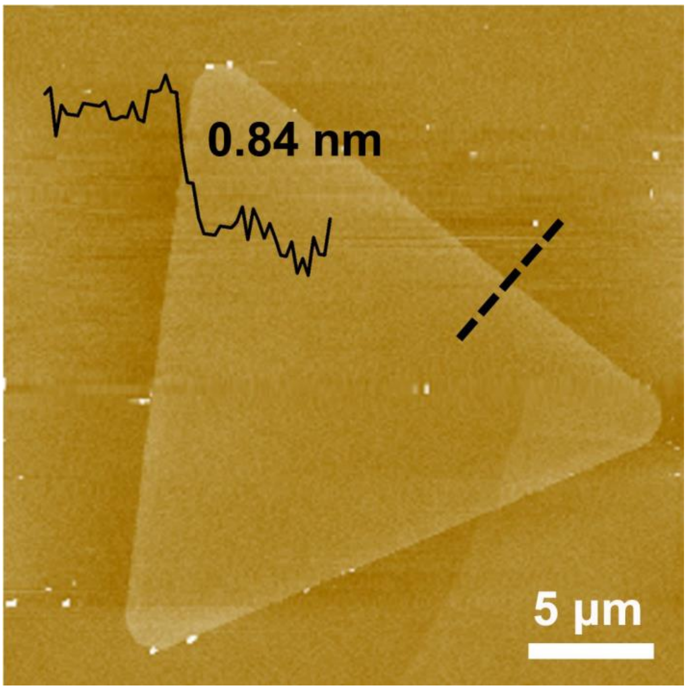

Supplementary Fig. 2 The AFM image of monolayer pristine MoS_2 and the corresponding height profile along the dashed line.

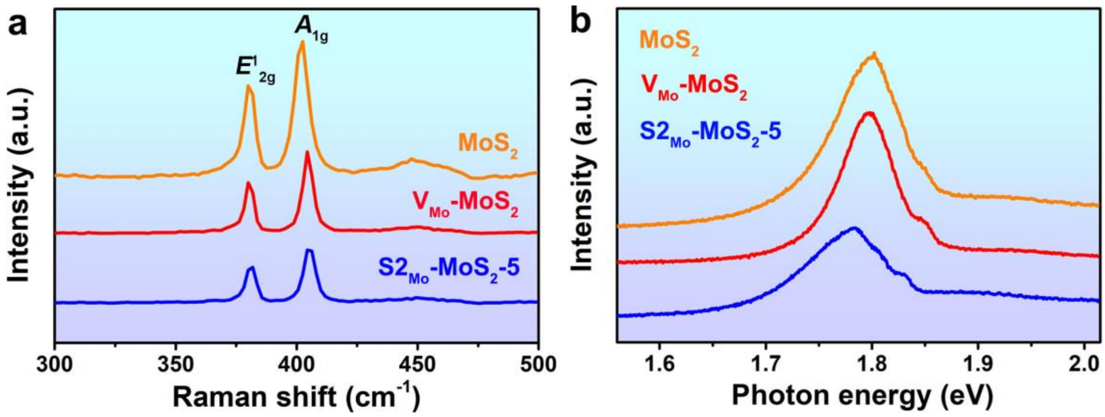

Supplementary Fig. 3 (a,b) The Raman and PL spectra of the three monolayer MoS_2–based materials.

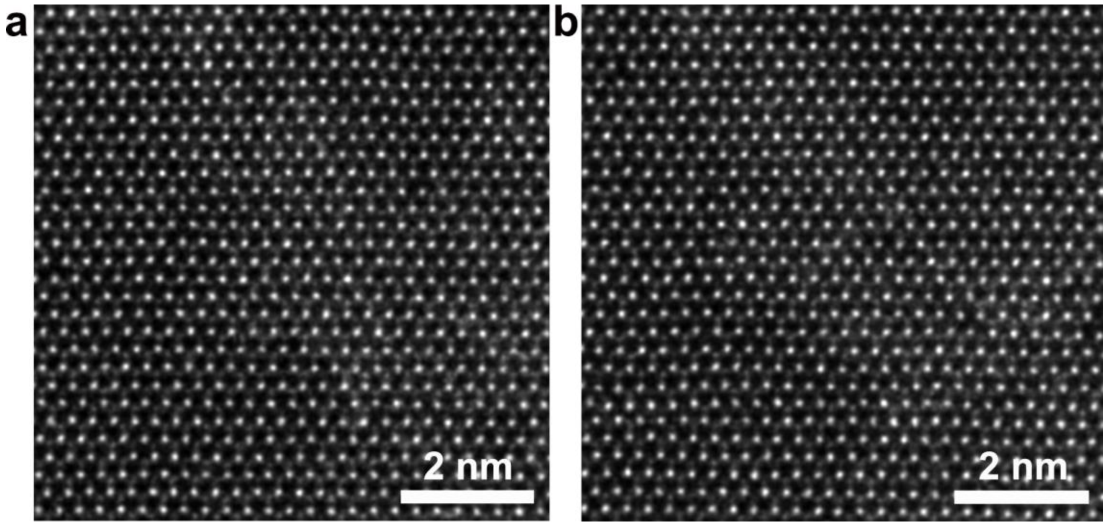

Supplementary Fig. 4 (a,b) Two HAADF images of monolayer MoS_2 in the same micro region were collected continuously under 300 kV and 15 pA conditions. The results showed that the atomic structure of monolayer MoS_2 is not significantly changed under electron beam radiation when HAADF images are collected.

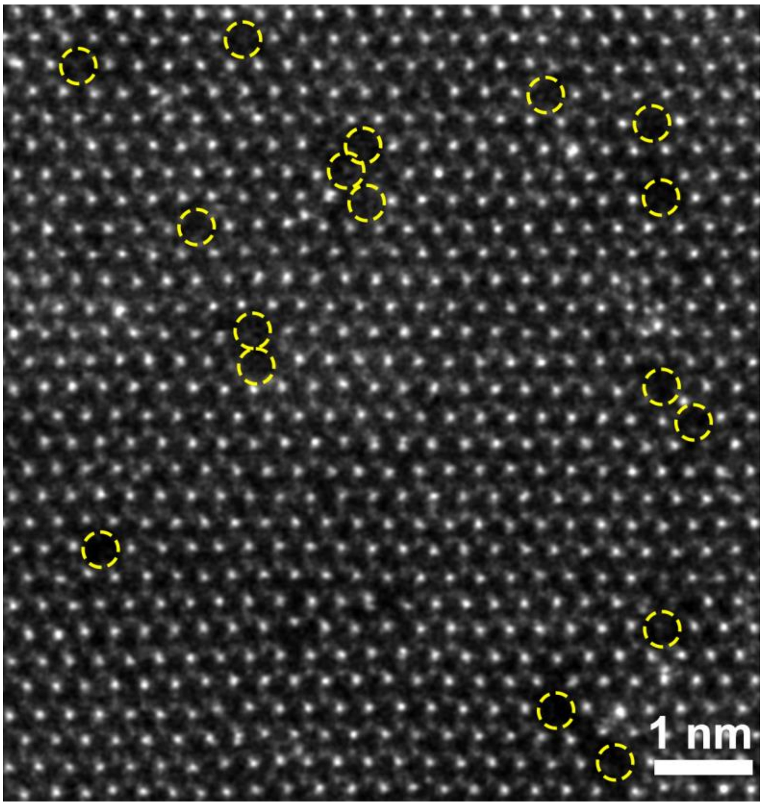

Supplementary Fig. 5 Larger-area atomic-resolution HAADF-STEM image of monolayer V_{Mo}-MoS_2-1. The yellow circles represent the Mo vacancies, and the overall average concentration of the Mo vacancies is approximately 2.5 %.

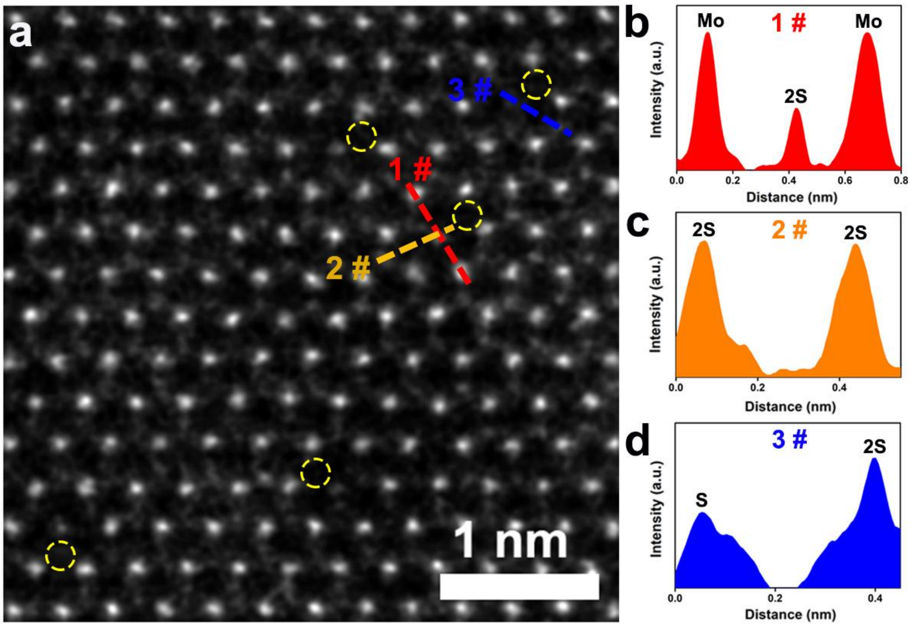

Supplementary Fig. 6 HAADF-STEM atomic intensity analysis of monolayer $S2_{Mo}$-MoS_2-5. (a) Atomic-resolution HAADF-STEM image of monolayer $S2_{Mo}$-MoS_2-5, which is the raw data of Fig. 1g. The yellow circles represent the Mo vacancies. (b-c) The atomic intensity diagrams of Mo atoms and two S atoms (inset #1), and two S atoms and two S atoms (inset #2). These results indicate that two S atoms occupy the Mo site in the antisite defect structure. (d) The atomic intensity diagrams of two S atoms and single S atoms (inset #3). This result indicates that there is also a single S atom occupying the Mo site in this antisite defect structure.

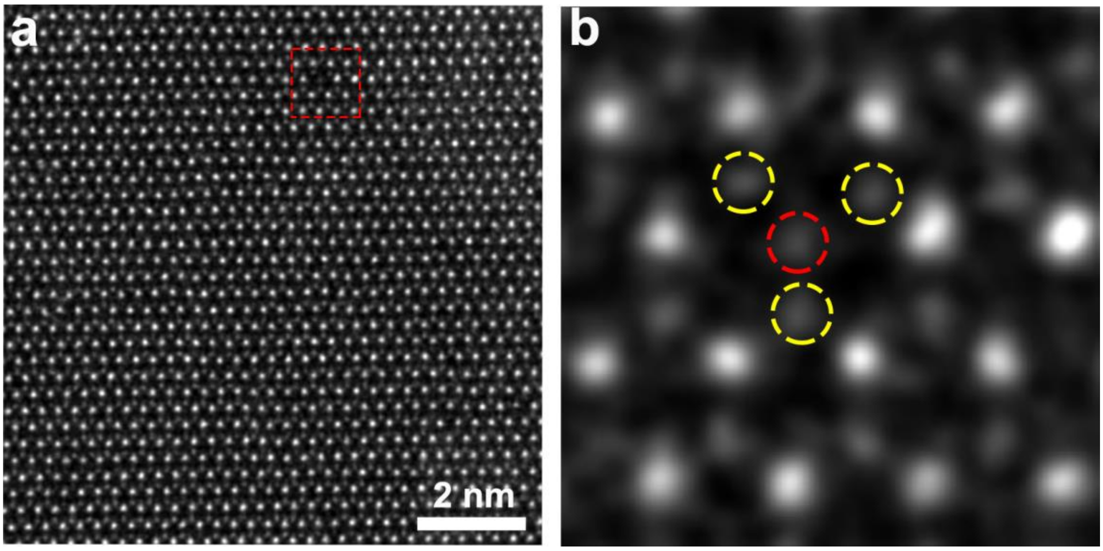

Supplementary Fig. 7 The atomic-resolution HAADF-STEM image of monolayer pristine MoS_2. (a) The existence of an antisite defect can be found in the red dashed region of pristine MoS_2. (b) The enlarged image corresponding to the red dashed area in (a). The red circle represents the antisite two S atoms, while each of the yellow circles represents two normal S atoms, indicating that six S atoms around the antisite defect remain in their original positions.

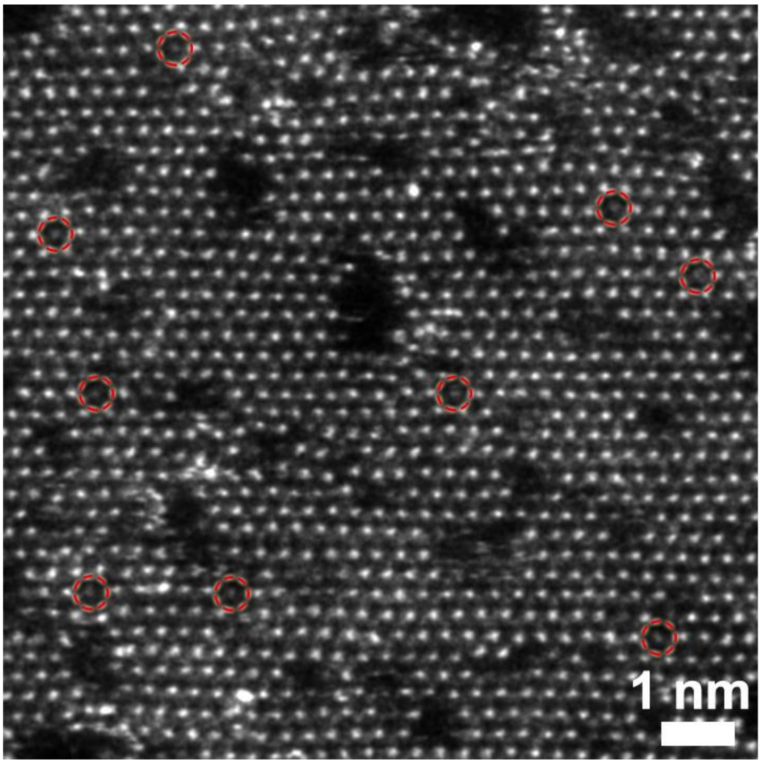

Supplementary Fig. 8 The atomic-resolution HAADF images of monolayer $S2_{Mo}$-MoS_2-15. In $S2_{Mo}$-MoS_2-15, some antisite defect structures are shown in red circles, and more hole structures have also been found. This shows that the atomic structure of monolayer MoS_2 will be obviously destroyed after calcination for 15 minutes at 400 °C.

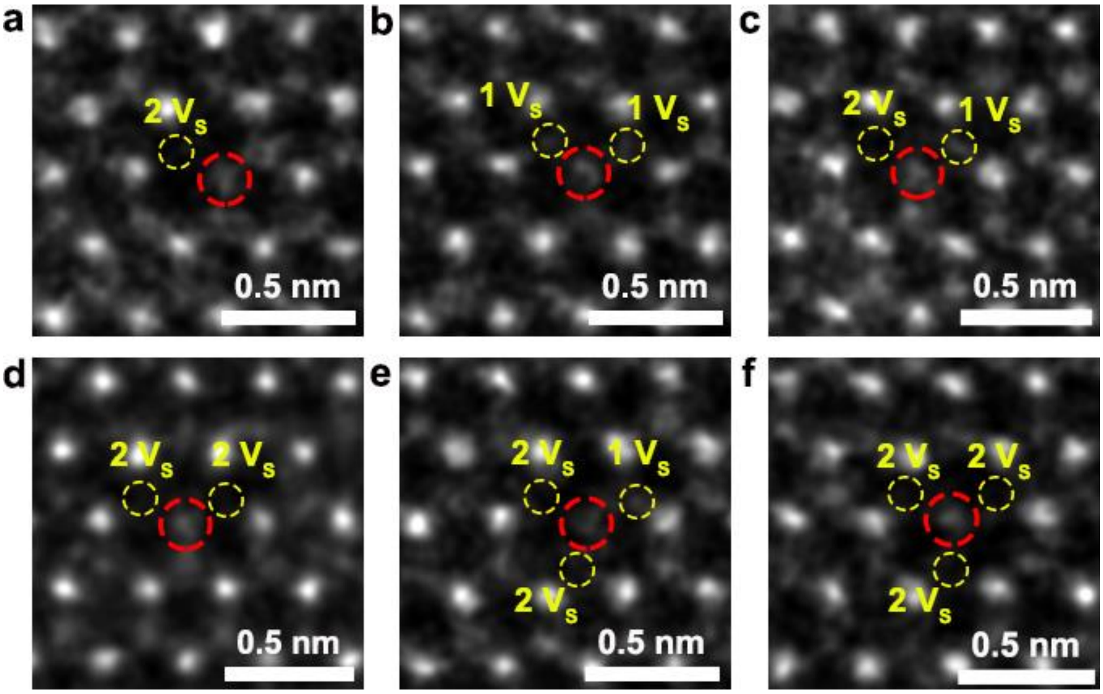

Supplementary Fig. 9 Atomic-resolution HAADF-STEM image of monolayer $S2_{Mo}$-MoS_2-5. (a-f) The distribution of different numbers of S vacancies (V_S) around the antisite defect structure in different regions, with the number of S vacancies ranging from 2 to 6.

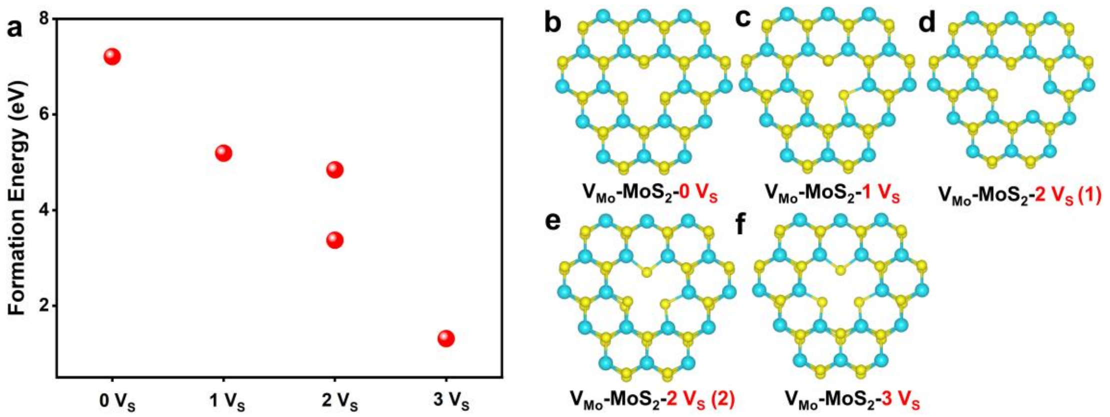

Supplementary Fig. 10 (a) The formation energy of Mo vacancies (V_{Mo}-MoS_2) with the increase of S vacancies (V_S). (b-f) The atomic structures of V_{Mo}-MoS_2 with different S vacancies.

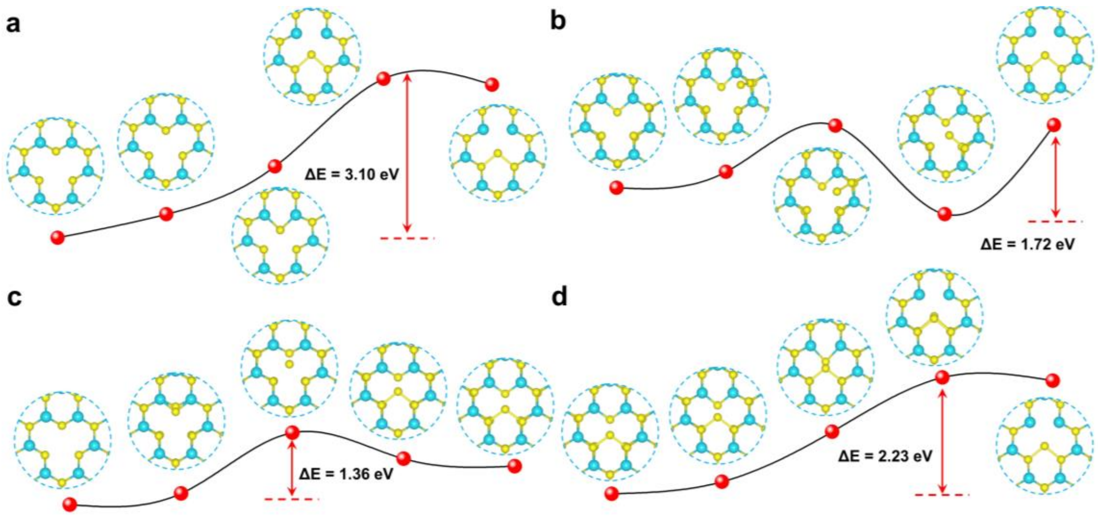

Supplementary Fig. 11 (a) The energy profile for simultaneous migration of two S atoms to occupy an Mo vacancy site to form an $S2_{Mo}$ antisite defect. (b) The energy profile of a special growth mechanism, in which an $S2_{Mo}$ antisite defect is formed by the aggregation of one S atom and another one free S atom escaping from the lattice. As shown in the atomic structures in the insets, along with the formation of Mo vacancies, one S atom migrates out of its original site and bonds with nearby free S atom to form an $S2_{Mo}$ antisite defect. The energy profiles clearly show that such growth process has a low energy barrier of ~ 1.72 eV, suggesting good kinetic feasibility. (c) The energy profile of one S atom migrating to form an intermediate S_{Mo} defect. (d) The energy profile of the further migration of an S atom to form an $S2_{Mo}$ defect from an already existing S_{Mo} defect.

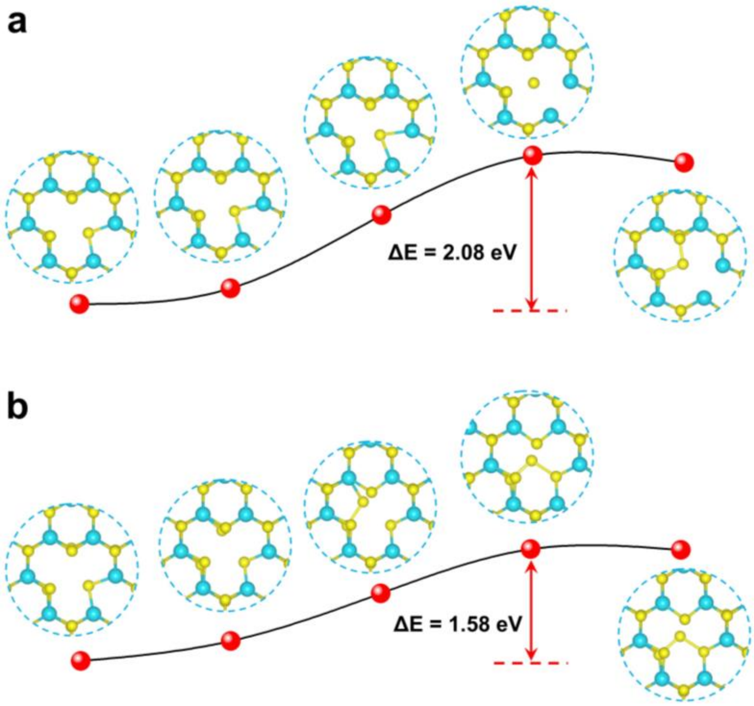

Supplementary Fig. 12 (a-b) The energy profiles for the formation of S_{Mo} antisite defect with the existence of one S vacancy (V_S).

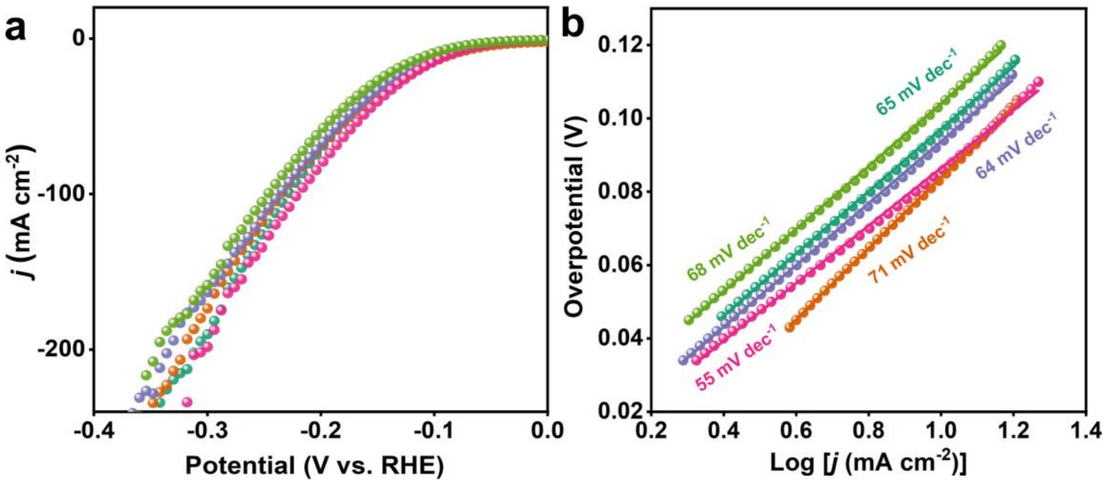

Supplementary Fig. 13 (a) Polarization curves and (b) Tafel plots of five 2D $S2_{Mo}$-MoS_2-5 samples. The results show that our electrochemical measurements are reproducible from the similar current density values and slopes.

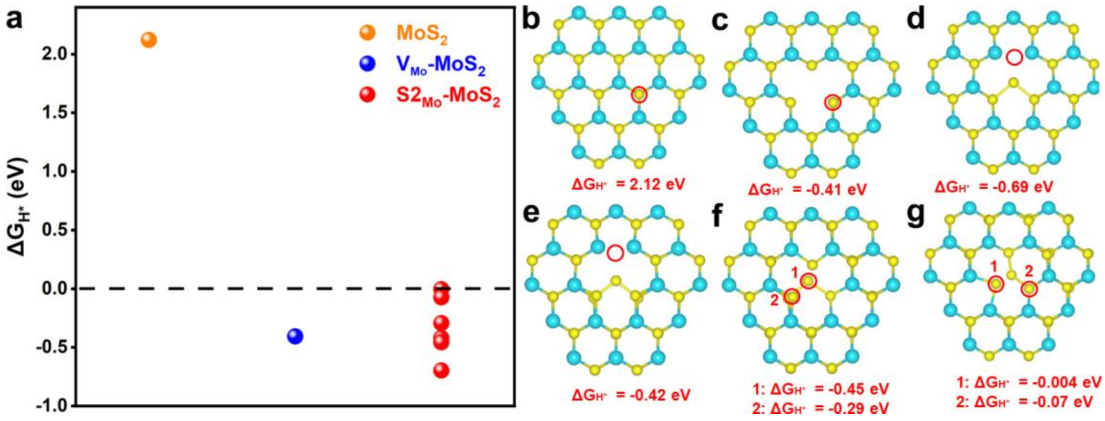

Supplementary Fig. 14 (a) Calculated ΔG_{H*} for MoS_2, V_{Mo}-MoS_2, $S2_{Mo}$-MoS_2 and antisite defect structures with S vacancies. (b-g) The corresponding atomic structures and detailed ΔG_{H*} values are also shown, and active sites are denoted by red circles. In addition, in terms of the active sites, for pristine MoS_2, the surface S atoms with saturated bonds are in an inactive state and exhibit catalytic inertness. Although the absence of Mo atoms can change the bonding properties of surrounding S atoms and activate them to become reaction active sites, the activated active sites are only limited to a small number of S atoms around the Mo vacancy. Compared to the Mo vacancy, antisite defects with various structures effectively modulate the atomic configurations and activate many different types of active sites, including exposed Mo atoms, antisite S atoms and surrounding S atoms of antisite defects.

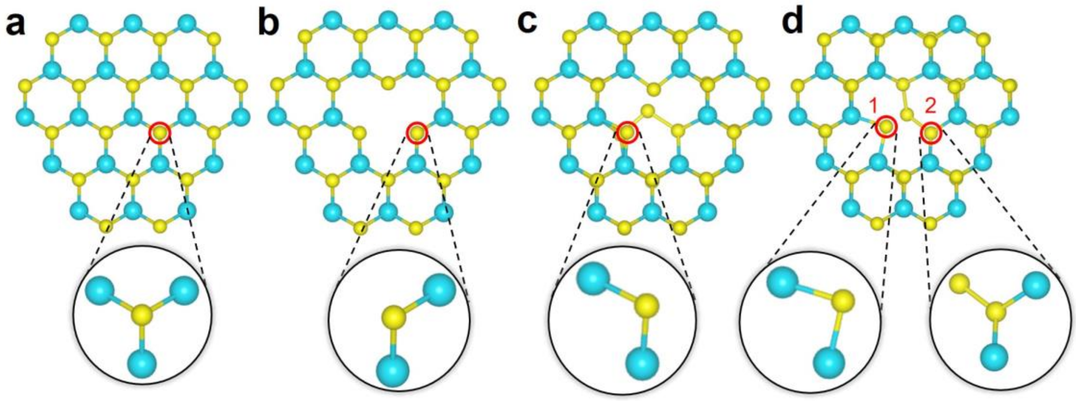

Supplementary Fig. 15 (a-d) The coordination demonstration of active S sites of pristine MoS_2, V_{Mo}-MoS_2, and $S2_{Mo}$-MoS_2.

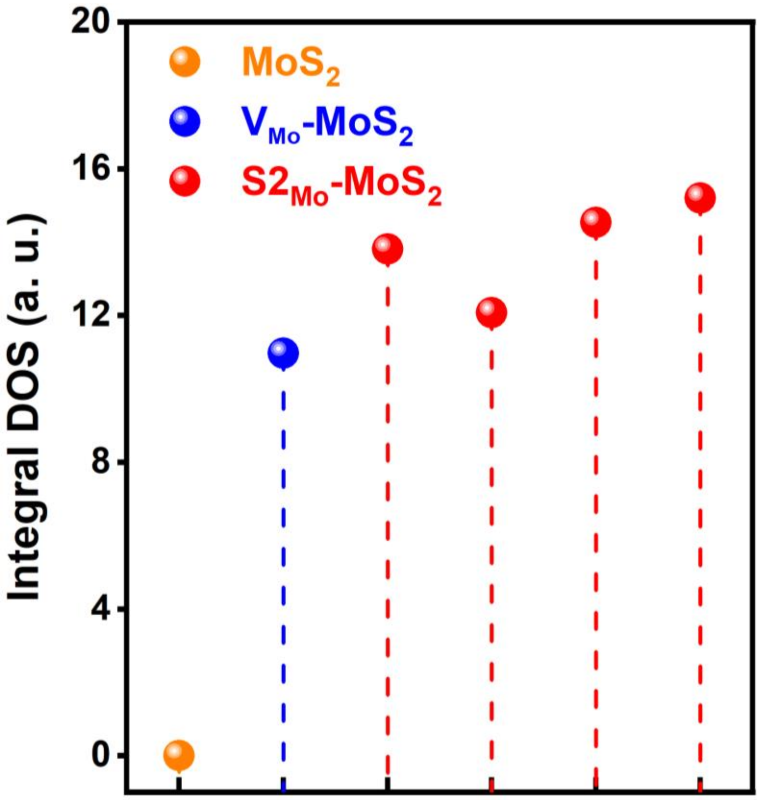

Supplementary Fig. 16 Integral DOS near the Fermi level for pristine MoS_2, V_{Mo}-MoS_2, $S2_{Mo}$-MoS_2, and antisite defect structures with S vacancies. The detailed atomic structures and corresponding DOS are shown in Supplementary Fig. 14.

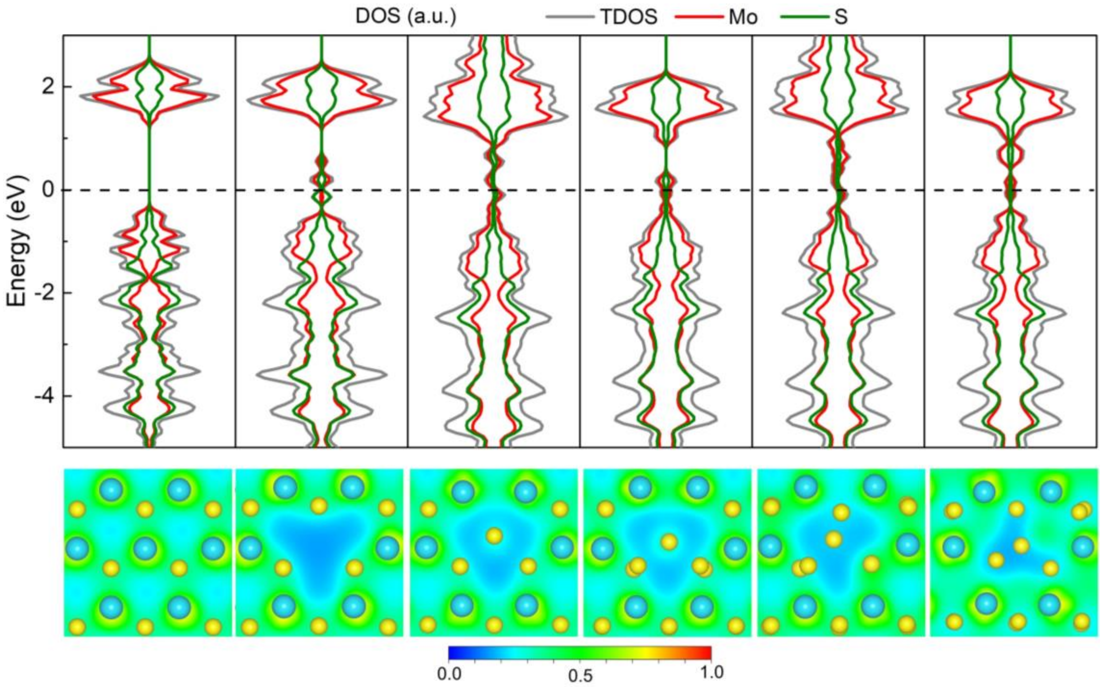

Supplementary Fig. 17 Density of states (DOS) and corresponding charge density distributions for pristine MoS_2, V_{Mo}-MoS_2, $S2_{Mo}$-MoS_2, S_{Mo}-MoS_2 and antisite defect structures with S vacancies. By definition, a region with a value of 1.0 denotes ideal charge accumulation, whereas a region with a value near 0.0 signifies a remarkably low charge density.

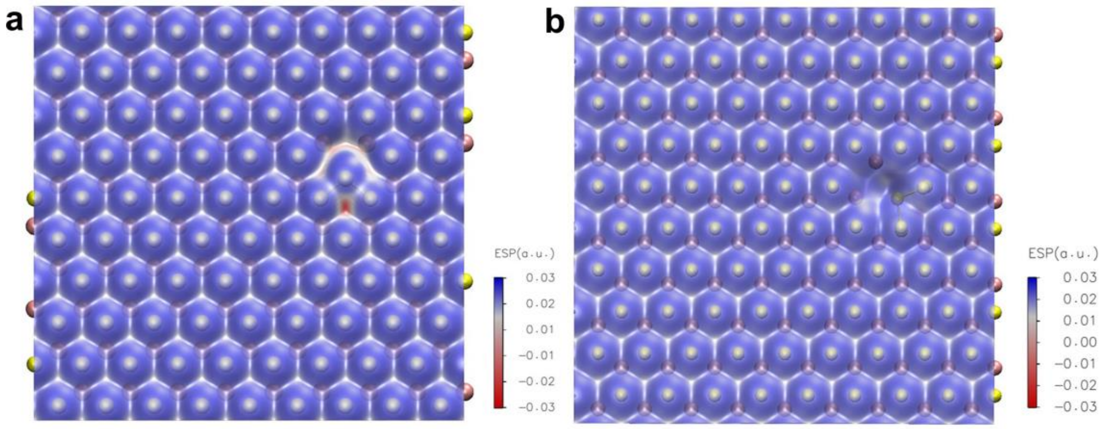

Supplementary Fig. 18 (a,b) Mapping Electrostatic Potential on electron density isosurface of two kinds of antisite defects. Red represents a low electrostatic potential, indicating that this region is easier to give electrons and more nucleophilic than other regions. While blue represents a high electrostatic potential, indicating that this region is easier to obtain electrons and more electrophilic than other regions. Calculations use the 0.001 electrons/bohr^3 density isosurface.

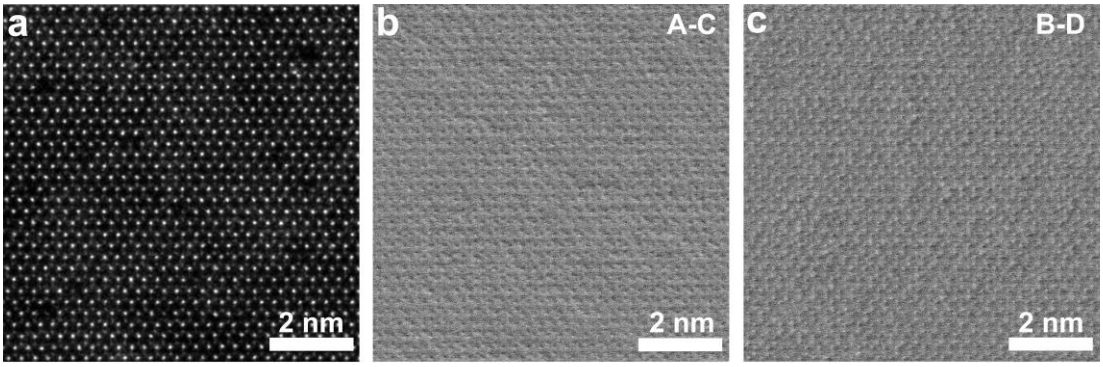

Supplementary Fig. 19 Atomic-resolution HAADF-STEM image and DPC images of monolayer $S2_{Mo}$-MoS_2-5. (a) The raw data of Fig. 4b, which is also the HAADF imaging area corresponding to the signal collected by the DPC segments detector. (b) The raw image obtained by subtracting the C segment signal from the A. (c) The raw image obtained by subtracting the D from the B.

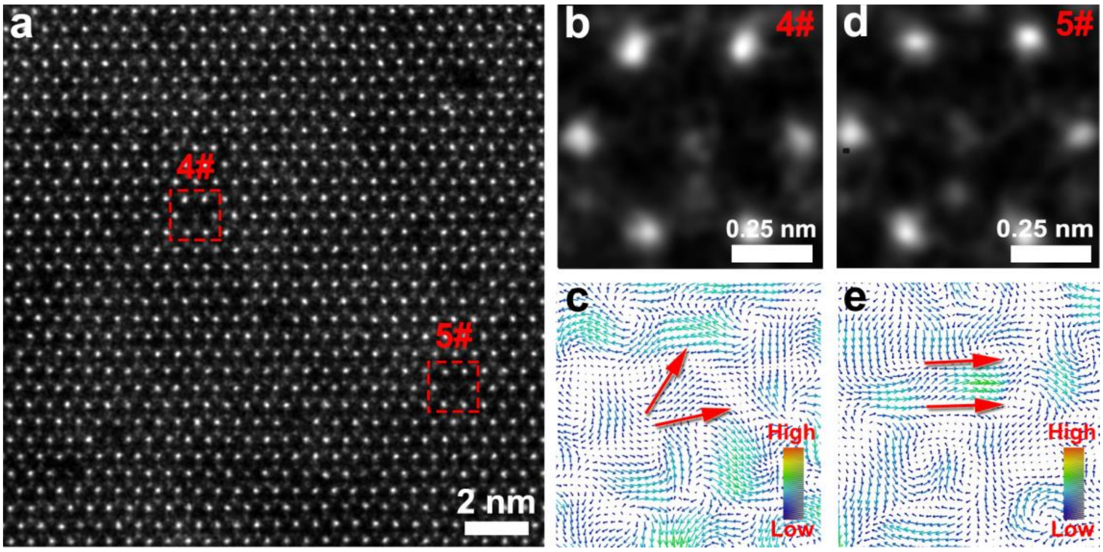

Supplementary Fig. 20 The HAADF-STEM image and corresponding atomic electric field distribution of $S2_{Mo}$-MoS_2-5. (a) The raw data of Fig. 4b. (b-c) The enlarged region of the antisite defect shown by 4# in (a) and the corresponding atomic electric field distribution. (d-e) The enlarged region of the antisite defect shown by 5# in (a) and the corresponding atomic electric field distribution. These results indicate that the different types of antisite defects from the type in Fig. 4d also exhibit polarized electric field distribution, as shown by the red arrows.

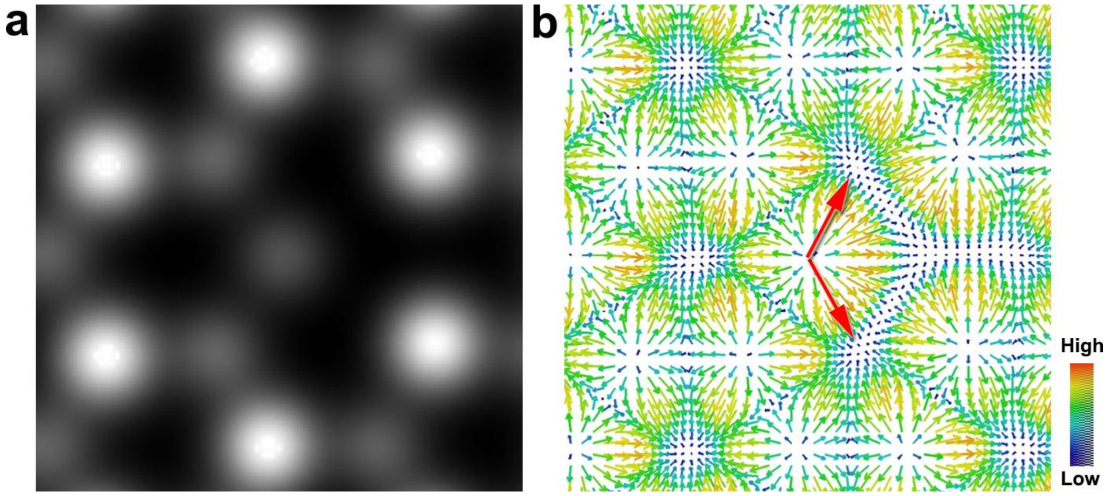

Supplementary Fig. 21 (a,b) Simulated HAADF image and corresponding DPC image of monolayer MoS_2 with the antisite defect structure. The red arrows in (b) indicated the electric field polarization region.

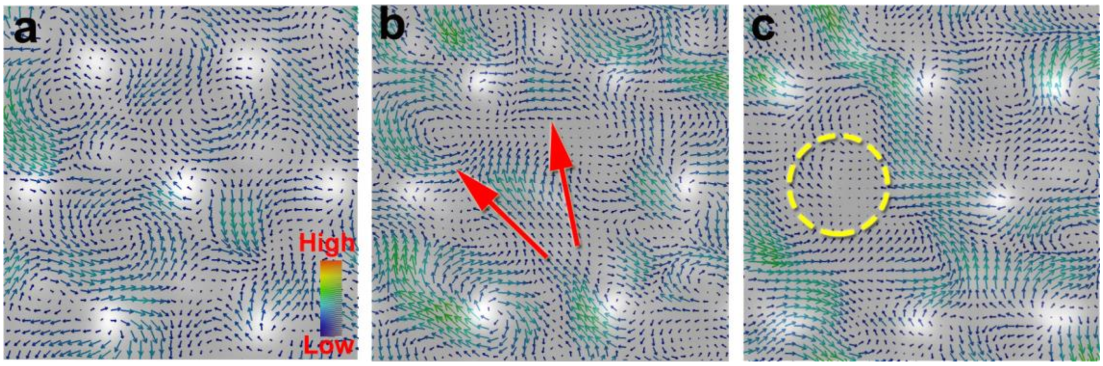

Supplementary Fig. 22 (a-c) The overlay images of HAADF-STEM and corresponding DPC map in Figure 4c-h of pristine atomic structure (a), the antisite defect structure (b) and Mo vacancy structure (c) in monolayer MoS_2.

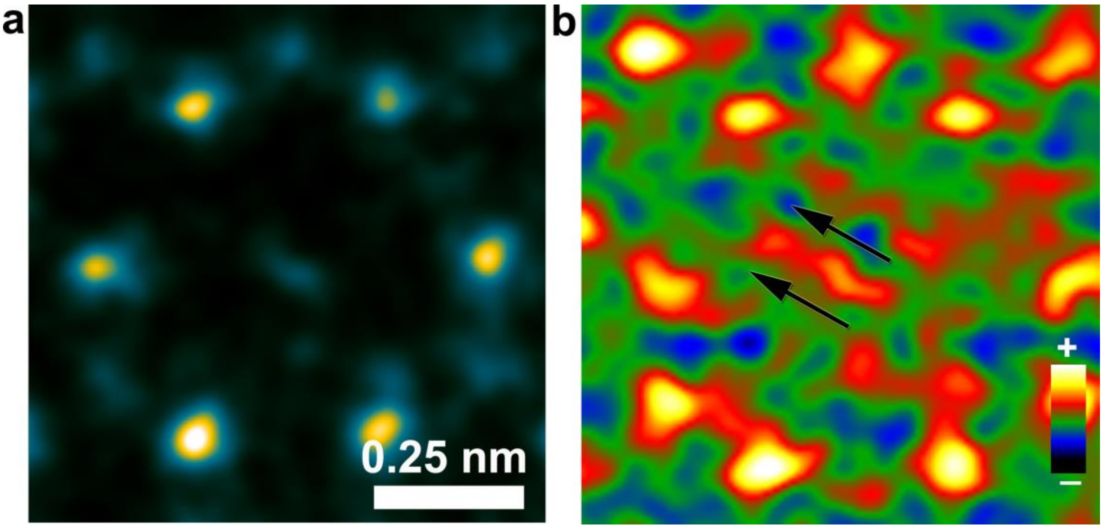

Supplementary Fig. 23 (a,b) HAADF-STEM image and corresponding dDPC image of $S2_{Mo}$-MoS_2-5. The HAADF image is the raw data of Figure 4d. The results show that the charge distribution of antisite defects structure was asymmetric, which is different from the elliptical charge distribution of the surrounding Mo atom. Notably, the accuracy of dDPC technology with four-segments detector may be difficult to distinguish the difference of charge density distribution of individual atoms.
